# Correction: Marin et al. Clinical Correlations between Serological Markers and Endometrial Cancer. *Cancers* 2024, *16*, 1935

**DOI:** 10.3390/cancers16203435

**Published:** 2024-10-10

**Authors:** Alina-Gabriela Marin, Alexandru George Filipescu, Răzvan Cosmin Petca, Radu Vlădăreanu, Aida Petca

**Affiliations:** 1 “Carol Davila” Faculty of Medicine, Department of Obstetrics and Gynecology, University of Medicine and Pharmacy, 050474 Bucharest, Romania; alina.marin@drd.umfcd.ro (A.-G.M.); alexandru.filipescu@umfcd.ro (A.G.F.); radu.vladareanu@umfcd.ro (R.V.); aida.petca@umfcd.ro (A.P.); 2 Department of Obstetrics and Gynaecology, Elias Emergency University Hospital, 011461 Bucharest, Romania; 3 Department of Urology, “Prof. Dr. Th. Burghele” Clinical Hospital, 20 Panduri Street, 050659 Bucharest, Romania

**Additional Affiliations**

In the published publication [1], there was an error regarding the affiliations for **Alina-Gabriela Marin** and **Alexandru George Filipescu**. In addition to affiliations “1,2”, there should also be “†”. The author contributions of the [1] also need to be updated, and the revised content is as follows. The authors apologize for any inconvenience caused and state that the scientific conclusions are unaffected. This correction was approved by the Academic Editor. The original publication has also been updated.
